# Felty's Syndrome as an initial presentation of Rheumatoid Arthritis: a case report

**DOI:** 10.1186/1757-1626-2-206

**Published:** 2009-11-18

**Authors:** Disaya Chavalitdhamrong, Ana Molovic-Kokovic, Andrey Iliev

**Affiliations:** 1 Department of Internal Medicine, James J Peters Veterans Affairs Medical Center, Mount Sinai School of Medicine Program, 130 W Kingsbridge Road, Bronx, NY 10468, New York, USA; 2 Department of Internal Medicine, Albert Einstein College of Medicine, 1300 Morris Park Avenue, Bronx, NY 10461, USA; 3 Department of Internal Medicine, North Central Bronx Hospital, New York City Health and Hospitals Corporation, 3424 Kossuth Avenue, Bronx, NY 10467, New York, USA

## Abstract

**Introduction:**

Felty's syndrome is an uncommon but severe extra-articular manifestation of rheumatoid arthtitis. Felty's syndrome is characterized by the triad of rheumatoid arthtitis, neutropenia, and splenomegaly. The lifetime risk of Felty's syndrome for a rheumatoid arthtitis patient is less than 1% and there are only few case reports of Felty's syndrome with neutropenia preceded clinical evidence of arthritis. We present a case which is atypical presentation of Felty's syndrome without arthritis.

**Case presentation:**

We present a case of 31-year-old man who presented with fever and skin infection, found to have neutropenia. The work up showed splenomegaly and other evidences support Felty's syndrome diagnosis without arthritis presentation.

**Conclusion:**

Patients with unexplained, continuous neutropenia without arthristis but with high level of rheumatoid factor and positive antibodies to cyclic citrullinated peptides should be suspected of developing Felty's syndrome as an initial presentation of rheumatoid arthtitis.

## Case presentation

31-year-old Caucasian male with no significant past medical history presented with fever of 102 F, chills and 4 centimeters painful redness induration at right inguinal region for 1 week. He denied chest pain, shortness of breath, palpitations, cough, headache, dizziness, nausea, vomiting, abdominal pain, bowel or urinary symptoms, weight or appetite changes. He has no drug use, sick contacts, recent travel, exposure to tuberculosis and unprotected sexual exposure. He does not smoke. He drinks beer occasionally on weekends but denies illicit drug use. He works in a food store with no direct raw food contact. He has no history of sexually transmitted diseases. His paternal aunt has Rheumatoid Arthritis (RA).

Physical examination demonstrated mild pallor, cellulitis at right inguinal area and left inguinal lymphadenopahy. Arthritis and rheumatoid nodules were absent.

Hemoglobin was 10.6 g/dL, hematocrit was 30% with MCV of 86.4, white blood cell count was 1.2 × 10^9^/L with absolute neutrophil count of 450 (0.45 × 10^9^/L) and platelet was 221 × 10^9^/L. Peripheral blood smear showed no significant abnormality with normal appearing white blood cells with few toxic granulations. Bone marrow biopsy showed normocellular and maturing trilineage hematopoiesis. Flow cytometry showed no evidence of lymphoproliferative disorder. He had negative tests for Syphilis, HIV, hepatitis B and hepatitis C. Monospot test, PPD skin test, ANA panel and Parvovirus B19 also were negative. Erythrocyte sedimentation rate was 50 mm/hr. C-reactive protein was 60.2 mg/l. Lymph node biopsy from left inguinal node revealed benign reactive lymph node. Abdominal computed tomography showed splenomegaly with triangular hypodense lesion at the lateral aspect of the mid portion of the spleen (abcess versus infarct). Negative Galium scan confirmed the diagnosis of splenic infarct. Rheumatoid factor (RF) was positive with titer of 1:640. Antibodies to cyclic citrullinated peptides (anti-CCP) were positive >250. Joint x-rays including wrists, hands, knees, ankles, feet showed no evidence of erosions.

The provisional diagnosis was Felty's Syndrome (FS). Treatment was initiated with methotrexate and granulocyte colony-stimulating factor (G-CSF). The neutropenia initially corrected and the erythrocyte sedimentation rate and C-reactive protein improved significantly. However, 2 weeks after cessation of G-CSF therapy the neutrophil count declined and second course of G-CSF was given. Overall treatment was tolerated well, except for the development of mild fever, mild arthritis and transient thrombocytopenia which are known side effects of G-CSF treatment. He currently remains on methotrexate with reasonable control of his symptoms.

## Discussion

RA is a chronic inflammatory arthritis with significant extra-articular manifestations. FS is a severe extra-articular feature of RA. FS is characterized by the triad of RA, neutropenia, and splenomegaly. The lifetime risk of FS for a RA patient is less than 1% [1]. Over 95% of FS patients are positive for RF with high titers [1,2]. FS usually develops after a long course of RA [2]. Arthritis almost always appears first and typically has been present for 10 years or more before neutropenia is recognized [3]. The articular disease in FS is usually severe in terms of both erosions and deformity [2]. In very rare cases, neutropenia appears before or with no arthritis and this patient is a representation of the latter [4-7].

In this case, the patient presented with skin infection in neutropenic setting. The most common infections affect the skin, mouth, and upper and lower respiratory tract [2]. Neutropenia and splenomegaly with elevated erythrocyte sedimentation rate, elevated C-reactive protein and anemia of chronic disease pointed toward connective tissue disorder. The clues that lead to the diagnosis of RA were high-titer RF and positive anti-CCP. Anti-CCP has very high specificity for RA-96% [8]. Combination of RF and anti-CCP has specificity of 99.5% for RA and highly predictive for development of erosions at 5 years of disease [8]. This case is an atypical presentation of FS because the lack of severe long lasting course of erosive RA. There is no specific diagnostic test for FS. It is a clinical diagnosis in RA with unexplained neutropenia and splenomegaly.

Patient was started treatment for presumed FS with methotrexate and G-CSF. Patient subsequently developed joint pain and swelling with less than 30 minutes stiffness in proximal interphalangeal joints, metacarpophalangeal joints, wrists, and knees. The non-specific arthritis was most likely due to the side effect of G-CSF as it disappeared after the medication was temporarily stopped. However, the patient needs to be followed closely, as repeat episode of arthritis in the future can be due to RA rather than G-CSF alone.

Treatment of neutropenia is mainly comprised of disease-modifying anti-rheumatic drugs (DMARDs) including methotrexate, hydroxychloroquine, auranofin, penicillamine, glucocorticoids, and G-CSF. The first choice for treating both neutropenia and arthritis is methotrexate which is safe, effective and well tolerated in these patients [9]. Recently, there has been an interest in the biologic agent rituximab in the treatment of FS but only a few cases has been reported [10]. Leflunomide, sulfasalazine and cyclophosphamide also have been reported but the experience is very limited [11]. The controlled studies of different treatment modalities are not available because of the rarity of this syndrome. Splenectomy produces a long-term hematologic response in 80% of patients but is usually reserved at the end of the treatment algorithm for treatment-resistant cases. [12].

G-CSF has no effect on the activity of RA but is effective and generally well-tolerated for the treatment of neutropenia due to FS [13]. Quick improvement in neutropenia has been reported with G-CSF [14]. G-CSF has been used as treatment for FS but with the known side effects of fever, thrombocytopenia and arthritis, all of which this patient had after receiving G-CSF [13]. The sustained granulopoietic response has been reported in some cases but the neutrophil count often declines when growth factor treatment is stopped but generally stabilized at a level that exceeded the pretreatment count [13]. Patients who tolerate G-CSF and have good hematologic responses may be candidates for prolonged therapy. This patient has been receiving G-CSF for maintenance of white blood cell count.

## Consent

Written informed consent was obtained from the patient for publication of this case report. A copy of the written consent is available for review from the journal's Editor-in-Chief.

## Competing interests

The authors declare that they have no competing interests.

## Authors' contributions

DC and AM wrote the manuscript and performed the literature search. AI reviewed the manuscript for intellectual content. All authors have read and approved the final manuscript.
